# Hyperuricemia and the gut microbiota: current research hotspots and future trends

**DOI:** 10.3389/fmicb.2025.1620561

**Published:** 2025-08-14

**Authors:** Jingjing Yang, Jing Chen, Dingxiang Li, Qin Wu, Yanan Zhang, Yujia Li, Yihui Deng

**Affiliations:** ^1^School of Integrated Chinese and Western Medicine, Hunan University of Chinese Medicine, Changsha, China; ^2^Key Laboratory of Hunan Province for Integrated Traditional Chinese and Western Medicine on Prevention and Treatment of Cardio-Cerebral Diseases, Hunan University of Chinese Medicine, Changsha, China; ^3^Department of Endocrine, Yueyang Traditional Chinese Medicine Hospital, Yueyang, China; ^4^School of Traditional Chinese Medicine, Hunan University of Chinese Medicine, Changsha, China

**Keywords:** gut microbiota, hyperuricemia, CiteSpace, VOSviewer, Bibliometrix, bibliometric, trends, hotspots

## Abstract

**Background:**

Hyperuricemia (HUA), found widely in humans and birds, is a key physiological factor responsible for the development of gout. In recent years, the relationship between the gut microbiota and HUA has garnered significant attention from researchers. This study aims to explore the current research hotspots, knowledge gaps, and future research trends regarding the gut microbiota and HUA.

**Methods:**

We performed a thorough search of the literature on gut flora and HUA published between 2005 and 2024 using the Web of Science and PubMed databases. The resulting data were analyzed using VOSviewer, CiteSpace, and Bibliometrix.

**Results:**

Including 735 papers in total, the study found that the number of publications in the subject increased significantly between 2020 and 2024, with 2024 being the year with the highest number of publications. The primary research countries are highlighted as China and the United States, with institutions such as the University of California, San Diego, and Qingdao University making significant contributions. Sanjay K. Nigam and Chenyang Lu have made the most important contributions as authors. Keywords analysis highlighted high-frequency terms including “gastrointestinal microbiome,” “uric acid,” “hyperuricemia,” “inflammation,” “gout,” and “probiotics.” In the visualization map of the keyword timeline, emerging research hotspots include “diets,” “dietary fiber,” “fecal microbiota transplantation,” and “gut-kidney axis.”

**Conclusion:**

This study is the first to conduct a quantitative literature analysis in the field of gut microbiota in HUA, revealing that the core research hotspots include disease-related microbiota characteristics, probiotic therapy, microecological intervention, and the gut-distal target organ axis. The emerging hotspots focus on dietary supplementation, fecal microbiota transplantation (FMT) treatment strategies, and in-depth research on the above organ axes. Provide valuable guidance for future research directions.

## 1 Introduction

Hyperuricemia (HUA) is a metabolic disorder characterized by elevated levels of uric acid (UA) in the bloodstream that exceed the normal physiological range (Li et al., 2020). UA, the final product of purine metabolism, is typically regulated by the body through excretion via the kidneys and intestines (Fathallah-Shaykh and Cramer, 2014). Elevated blood levels of UA, resulting in HUA, can occur due to increased production or decreased excretion. The global rise in HUA incidence, driven by lifestyle changes and the westernization of diets, has emerged as a significant public health issue. Notably, global research indicates that while the prevalence of HUA differs among regions and ethnicities, it is generally on the rise (Dehlin et al., 2020). The prevalence rates of HUA among adults in the United States, Finland, Australia, South Korea, and French Polynesia are reported to be 20.1%, 48%, 16.6%, 11.4%, and 71.6%, respectively (Kim et al., 2018; Chen-Xu et al., 2019; Pathmanathan et al., 2021; Timsans et al., 2023; Pascart et al., 2024). Additionally, HUA is more common in males than females, with its incidence rising with age. Clinically, HUA is a direct contributor to urological conditions, such as gout, kidney stones, and renal insufficiency (Jordan et al., 2019; Narang et al., 2021). Moreover, it is linked to a heightened risk of cardiovascular diseases such as hypertension, coronary heart disease, atherosclerosis, and heart failure (Yu et al., 2017; Lee et al., 2019; Kimura et al., 2021; Zheng et al., 2024). HUA is also associated with insulin resistance and may contribute to the development of type 2 diabetes (Wan et al., 2016; Zhang Y. et al., 2023). Therefore, the management and treatment of HUA are vital for enhancing patient quality of life and preventing associated complications.

The intestinal microbiota, a complex assemblage of microbial species in the human gut, is essential for the host’s metabolism and immune system, having co-evolved with humans for millennia (Thursby and Juge, 2017; Rinninella et al., 2019). The variety, homeostasis, and adaptability of the intestinal microbiota, as well as their mutualistic relationships with the host, have been influenced by a prolonged co-evolutionary process that dictates the complicated relationships between the gut microbiota and the health of the host (Luckey, 1972). The host benefits from various metabolic functions provided by the gut microbiota, which arise from the anaerobic fermentation of undigested dietary elements, like short-chain fatty acids (SCFAs), or metabolic products originating from both the microbes and the host (Clemente et al., 2012; Sun et al., 2025). Research has shown that changes in gut microflora composition and function are associated with the onset of metabolic diseases, including obesity, diabetes, and cardiovascular disease (Xu et al., 2024). The connection between gut flora and HUA has recently attracted considerable interest from researchers and healthcare professionals (Zhou X. et al., 2024). Research demonstrates that the gut flora (such as *Firmicutes* and *Actinobacteria*) can metabolize UA into xanthine or SCFAs (Liu et al., 2023). Additionally, dysbiosis within the gut microbiota may compromise intestinal barrier integrity and increase permeability, which in turn can influence UA excretion (Xu et al., 2019; Chen et al., 2020; Wang et al., 2022).

Researchers worldwide have made significant strides in exploring the connection between gut microbiota and HUA in recent years. Nonetheless, there have been no significant reports of bibliometric analysis or visualization studies in this field. The trajectory and progress of research on HUA and gut microbiota can be revealed through bibliometric analysis at a macro level. Consequently, we carried out a bibliometric analysis with the help of VOSviewer, CiteSpace, and Bibliometrix to investigate the research trends and emerging areas in the gut microbiome and HUA, thus making clear the themes that typify the junction of these two crucial research areas.

## 2 Materials and methods

### 2.1 Data collection

We searched PubMed and the Web of Science Core Collection (WOS) for all studies on the gut microbiota and HUA published between 1 January 2005, and 31 December 2024. On 15 February 2025, we completed all searches to prevent bias in the quantity of documents resulting from database upgrades (Zhu et al., 2025). Using the terms “gastrointestinal microbiomes,” “hyperuricemia,” and “uric acid” (as well as their MeSH synonyms), and adding a few more phrases that have been reported to be associated with the gastrointestinal microbiome (Yang et al., 2022), we expanded the scope of the searches. The terms for hyperuricemia and gut microbiota are provided in Supplementary Appendix 1.

### 2.2 Inclusion and exclusion criteria

**FIGURE 1:**
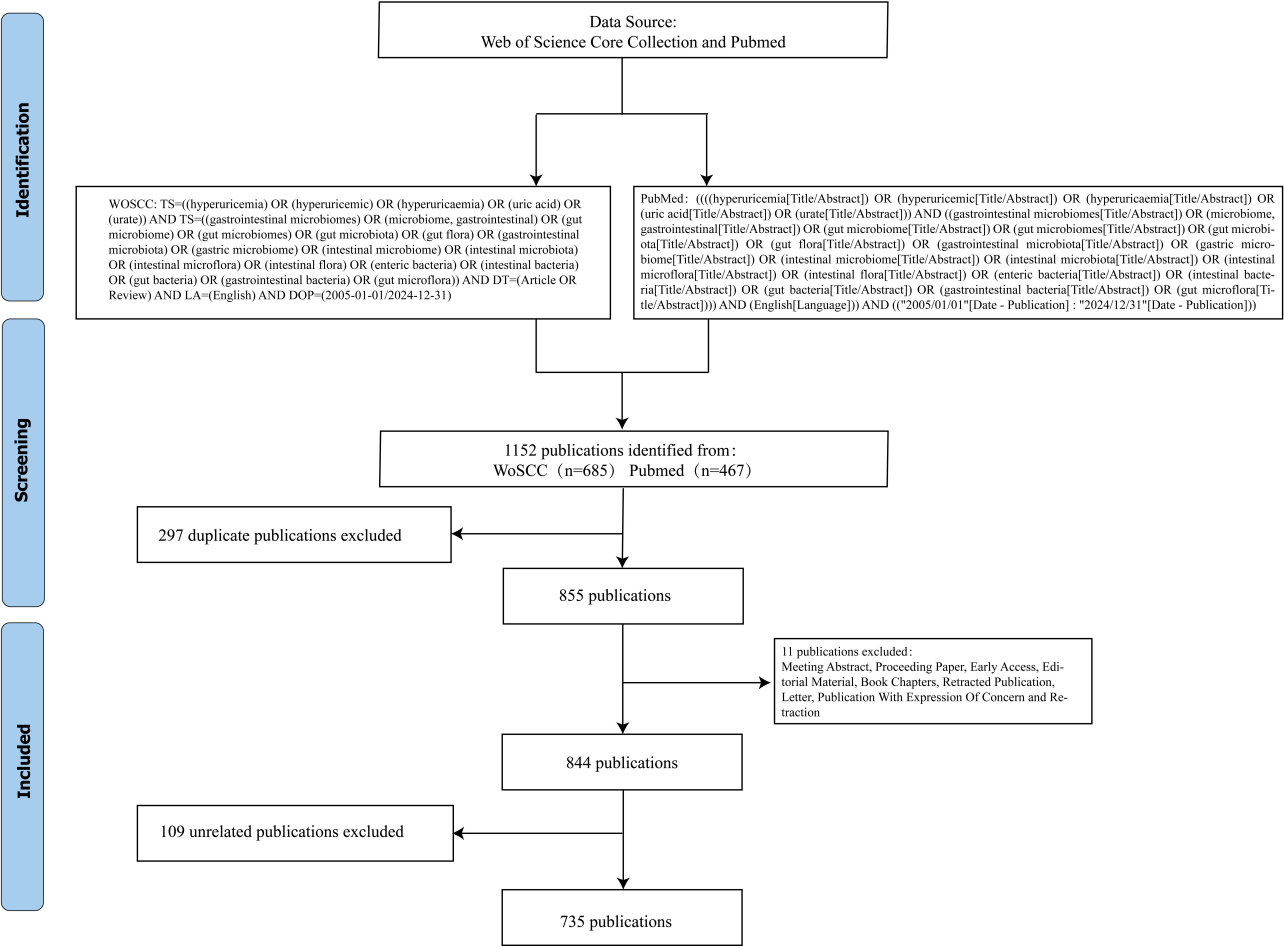
Flowchart for data retrieval and filtering.

Inclusion criteria: All original articles and reviews in English related to “intestinal flora” and “HUA.” Exclusion criteria: Duplicated literature, literature not related to “intestinal flora” and “HUA” research, as well as meeting abstracts, proceeding papers, early access, editorial material, book chapters, retracted publications, and letters. In this study, Jingjing Yang and Jing Chen independently screened and excluded studies. Any discrepancies that arose were resolved by Dingxiang Li, who made the final decisions. A total of 735 papers were included, including 130 reviews and 605 original articles. Figure 1 illustrates the flowchart of the literature search and screening process.

### 2.3 Data analysis and visualization

The export of the qualifying publications “Full Record and Cited References” is performed in either “Plain Text File” format, with the filename “download*.txt.” The “Plain Text File” was loaded into VOSviewer, CiteSpace, and Bibliometrix^1^ for graphing. Before analysis, synonymous terms were unified using “thesaurus_terms.txt” in VOSviewer 1.6.19 to create thesaurus files. This process included synonyms (e.g., “gut microbiome” and “gastrointestinal microbiome”), singular and plural forms (e.g., “broiler chicken” and “broiler chickens”), and different expressions (e.g., “xanthine oxidase” and “Xanthine oxidase enzyme”). Excel 2021 software produces tables for sorting various counts. We used VOSviewer 1.6.19 to summarize the leading authors, co-cited authors, countries/regions, institutions, journals, co-cited references, keywords, and associated knowledge maps. For the dual-map overlay of journals, we employed CiteSpace V (version 6.3 R3, downloaded from https://citespace.podia.com/). The settings for CiteSpace V were configured as follows: cluster labels (12), journal labels (8), arcs α (3), citing journal titles (min pubs: 10), and cited journal titles (min cites: 10).

## 3 Results

### 3.1 Publication and citation trends

**FIGURE 2:**
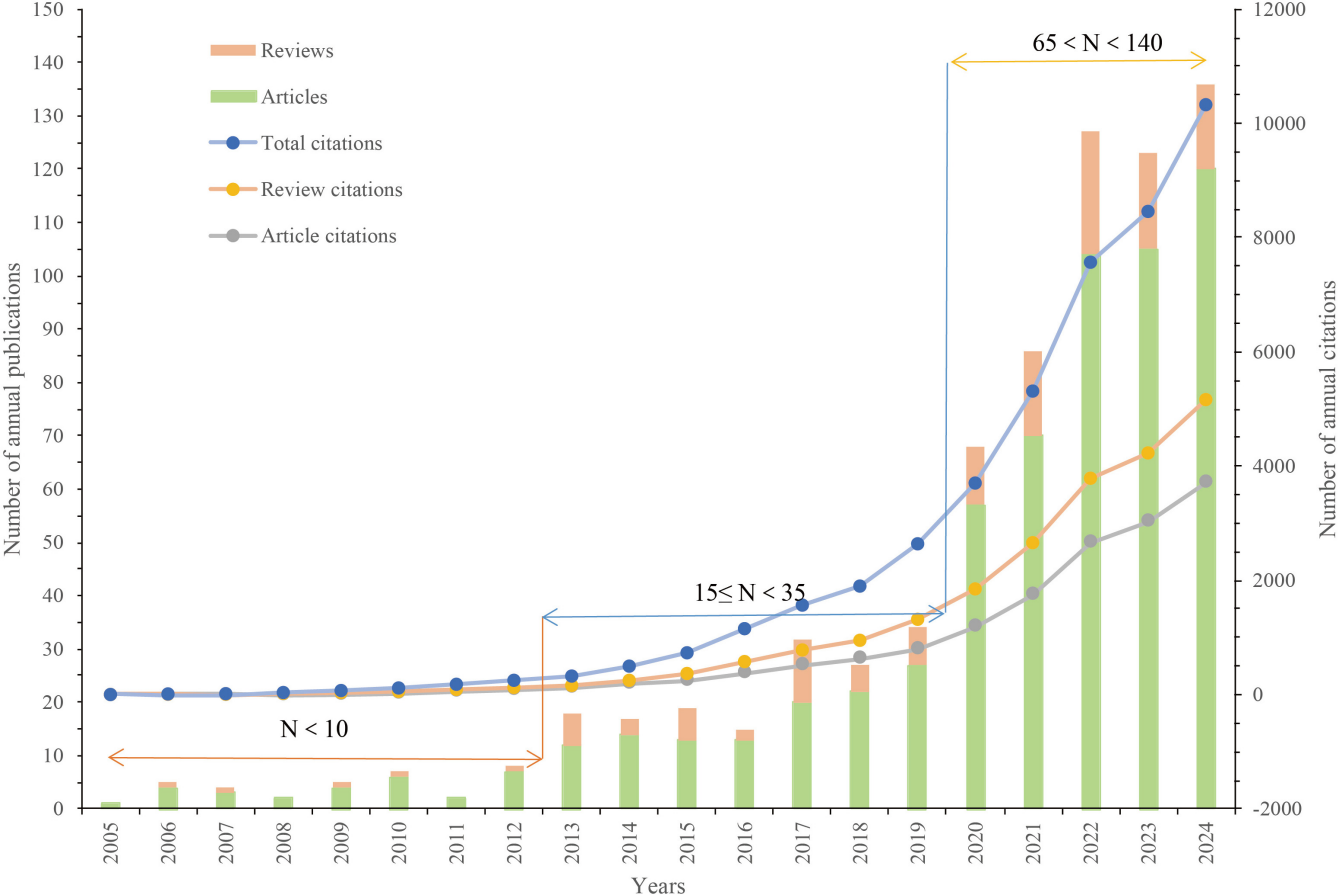
Trends in publications and annual citations for research on the gut microflora and hyperuricemia from 2005 to 2024.

A total of 735 publications addressing the gastrointestinal microbiome and HUA were analyzed for this research; 130 of these were reviews, while the remaining 605 were articles. Figure 2 displays the trends in publications and annual citations for research on the gut microflora and hyperuricemia from 2005 to 2024. The bar graphs represent the number of publications, whereas the line graphs represent annual citations. Figure 2 illustrates that between 2005 and 2012, fewer than 10 papers were published annually in the area of gut flora and HUA, reflecting a low level of research activity. From 2013 to 2019, there was a slow growth in the number of publications, with 15–35 papers published each year, and a corresponding increase in citations. Notably, there was a remarkable increase in the number of publications in the field from 2020 to 2024, indicating that the interest of researchers in exploring the link between gut microbiota and HUA is growing, with 2024 emerging as the peak year for publications. This trend demonstrates that the study of gut microbiota and HUA has received increasing attention over the past 5 years and is expected to continue to be a key area of research.

### 3.2 Countries/regions and institutions

**FIGURE 3:**
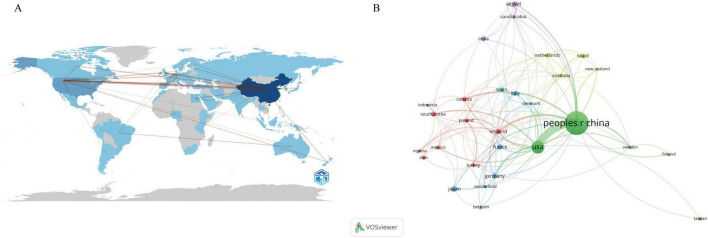
**(A)** The global publications’ geographic distribution map. **(B)** The global publications’ nations/regions collaboration map for the intestinal microbiota and hyperuricemia.

A collective of 72 distinct countries/regions participated in the study of the gastrointestinal microbiome and HUA. China had the highest number of publications (*n* = 413), followed by the United States (*n* = 126), Japan (*n* = 24), Italy (*n* = 21), and Germany (*n* = 20) (Table 1). According to Table 1, the United States has the highest total number of citations (*n* = 6,591), which is much higher than the number of citations for China (*n* = 5,200) and other high-output countries. Figure 3 displays the international collaboration networks of nations/regions. This map includes 30 countries/regions, each having published at least five articles. The size of the nodes represents the volume of publications, with larger nodes indicating a higher number of publications. Nodes sharing the same color denote a cluster with significant collaboration. The arcs illustrate the cooperation between different countries or regions, with thicker arcs signifying stronger ties. The figure reveals that both China and the United States have collaborative relationships with several countries, with the most frequent collaboration between China and the United States.

**TABLE 1:** Top 10 countries/regions and institutions investigating the intestinal microbiota and hyperuricemia.

Rank	Countries/regions	Documents (*N*)	Citations	Institutions	Documents (*N*)	Location	Citations
1	China	413	5,200	University of California, San Diego	25	United States	1,642
2	United States	126	6,591	Qingdao University	18	China	263
3	Japan	24	929	Zhejiang University	17	China	497
4	Italy	21	908	China Agricultural University	15	China	502
5	Germany	20	890	South China Agricultural University	15	China	91
6	France	19	533	Zhengzhou University	14	China	284
7	Egypt	18	175	China Agricultural University	12	China	169
8	Brazil	17	922	Ningbo University	11	China	143
9	Canada	17	784	Sun Yat-sen University	11	China	179
10	South Korea	17	795	Zhejiang Chinese Medical University	11	China	272

**FIGURE 4:**
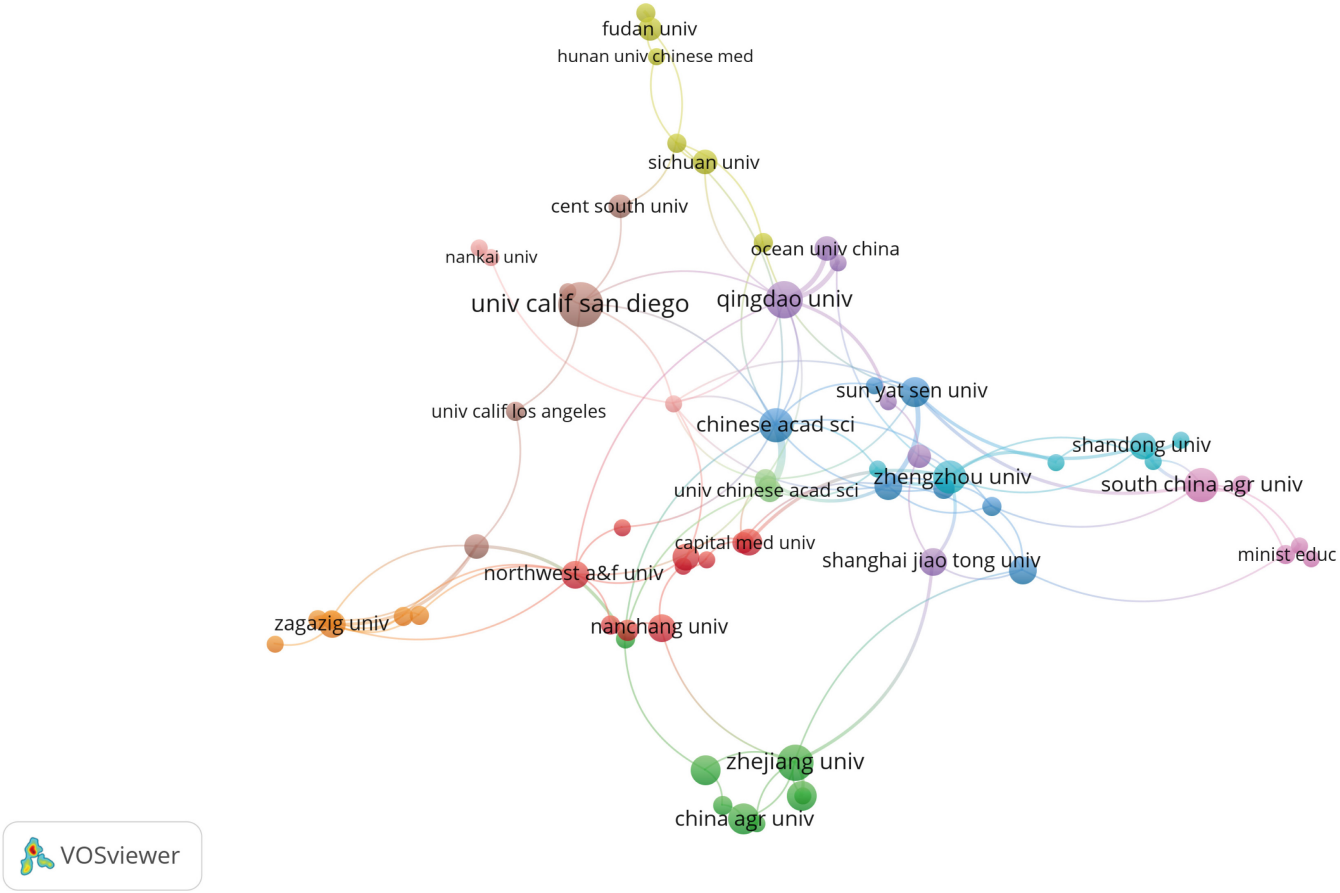
Collaborative network visualization of institutions involved in researching the intestinal microbiota and hyperuricemia.

A total of 1,265 institutions researched gut microbiota and HUA. The top 10 institutions with the highest output published a total of 149 articles (Table 1). The University of California, San Diego (UCSD) ranked first in terms of the number of publications and citations, with 25 articles. Qingdao University and Zhejiang University followed with 18 and 17 articles, respectively. Notably, 9 of the top 10 institutions in terms of publication volume are from China. Figure 4 shows the cooperative network among different institutions. The size of a node indicates the number of documents published by the institution. The larger the node, the more documents are published. The more curves there are, the more cooperative institutions there are; the thicker the curve, the closer the cooperation among the institutions. Figure 4 illustrates that the Chinese Academy of Sciences has the highest number of collaboration linkages, indicating that this institution has the most extensive collaborative network. In terms of international collaboration, the UCSD has engaged in close collaborations with top-ranking institutions, such as Qingdao University and the Chinese Academy of Sciences.

### 3.3 Authors and co-cited authors

A total of 4,622 authors participated in studies on HUA and the gut flora (Table 2). Sanjay K. Nigam from the University of California, San Diego, was the top contributor, publishing 19 papers. Subsequently, Chenyang Lu and Xiurong Su from Ningbo University each published nine papers. Additionally, we analyzed the number of co-citations among the authors. Most of the top 10 co-cited authors were American. Nicola Dalbeth, Jing Wang, Zhuang Guo, Sanjay K., and Richard J. Johnson, with more than 80 co-citations. Nigam was among the top five for co-cited. Notably, Sanjay K. Nigam ranked highly in both co-cited and publication output. Supplementary Figure 1 provides a visual representation of the analysis results, highlighting the associations among countries, affiliations, and authors.

**TABLE 2:** Top 10 authors with high productivity and co-citation in studies on the gut microflora and hyperuricemia.

Rank	Author	Counts	Institution	Co-cited authors	Citations	Institution
1	Sanjay K. Nigam	19	University of California, San Diego	Nicola Dalbeth	140	University of Auckland
2	Chenyang Lu	9	Ningbo University	Jing Wang	120	Zhejiang University
3	Xiurong Su	9	Ningbo University	Zhuang Guo	115	Ministry of Education of the People’s Republic of China
4	Kevin T. Bush	8	University of California, San Diego	Sanjay K. Nigam	89	University of California, San Diego
5	Jiaojiao Han	8	Ningbo University	Richard J. Johnson	83	University of Colorado, Renal Diseases and Hypertension
6	Zhixing He	8	Zhejiang Chinese Medical University	Yu Wang	76	Shaanxi Normal University
7	Yan Wang	8	Peking University	N. Yamaoka	73	Teikyo University
8	Chengping Wen	8	Zhejiang Chinese Medical University	Nosratola D. Vaziri	72	University California, Irvine
9	Jun Zhou	8	Ningbo University	Peter J. Turnbaugh	67	Washington University
10	Tiejuan Shao	8	Zhejiang Chinese Medical University	Patrice D. Cani	67	University Catholique de Louvain

### 3.4 Journal analysis

Research papers on gut microbiota and HUA have been published in 369 different journals. Table 3 lists the top 10 journals ranked by the number of articles and co-cited counts. The journal Nutrients published the highest number of research articles, totaling 24, followed by Frontiers in Microbiology and Food & Function, which published 21 and 18 articles, respectively. Furthermore, all 10 journals with the highest citation counts have been cited more than 350 times, with PLoS One leading the way with a total of 883 citations, followed by Scientific Reports and Nature, which have been co-cited 648 and 631 times, respectively. According to the overlay visualization of journal maps (Supplementary Figure 2), studies published in veterinary/animal science journals predominantly cited papers in molecular biology/genetics journals. Similarly, research studies published in Molecular, Biology, and Immunology journals cited papers in Environmental, Toxicology, and Nutrition journals, as well as in Molecular, Biology, and Genetics journals and Health, Nursing, and Medicine journals. Furthermore, studies published in Medicine, Medical, and Clinical journals primarily cited papers in Molecular, Biology, and Genetics journals and Health, Nursing, and Medicine journals.

**TABLE 3:** Top 10 journals and co-cited journals for research on the gut microflora and hyperuricemia.

Journals	Documents (*N*)	Citations	2023 IF	Co-cited journals	Co-citation	2023 IF
Nutrients	24	656	4.8/Q1	PLoS One	883	2.9/Q1
Frontiers in Microbiology	21	333	4.0/Q2	Scientific Reports	648	3.8/Q2
Food & Function	18	242	5.1/Q1	Nature	631	50.5/Q1
Frontiers in Nutrition	17	53	4.0/Q2	Nutrients	598	4.8/Q1
Scientific Reports	16	1,089	3.8/Q1	Poultry Science	592	3.8/Q1
Journal of Agricultural and Food Chemistry	15	150	5.7/Q1	Food & Function	476	5.1/Q1
PLoS One	15	704	2.9/Q1	Proceedings of the National Academy of Sciences of the United States of America	455	9.4/Q1
Frontiers in Cellular and Infection Microbiology	13	90	4.6/Q2	Journal of Biological Chemistry	446	4.0/Q2
Food Bioscience	12	27	4.8/Q1	Journal of Agricultural and Food Chemistry	399	5.7/Q1
Frontiers in Pharmacology	12	253	4.4/Q1	Frontiers in Microbiology	391	4.0/Q2

### 3.5 Reference analysis

**FIGURE 5:**
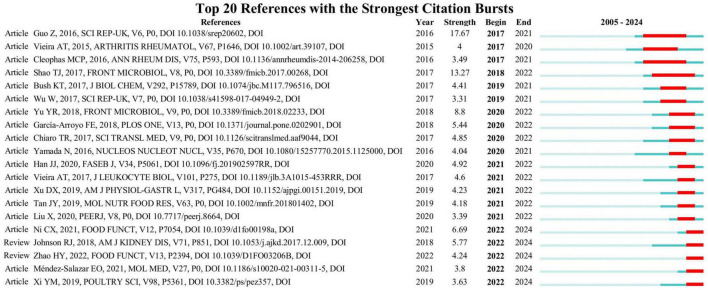
Top 20 references with the strongest citation bursts.

Citation burst analysis identifies significant and impactful literature from a specific time frame by pinpointing studies that experience a spike in citations during that period. Figure 5 shows the top 20 references with the strongest citation bursts, including 3 clinical studies, 15 experimental studies, and 2 reviews. The blue line depicts the timeline, and the red segments indicate the times when the references had bursts. Citation strength for the top 20 references spanned from 3.55 to 17.67. The citation explosion in this field began in 2017. Furthermore, two reviews and three articles are presently experiencing a burst.

### 3.6 Keyword analysis

**FIGURE 6:**
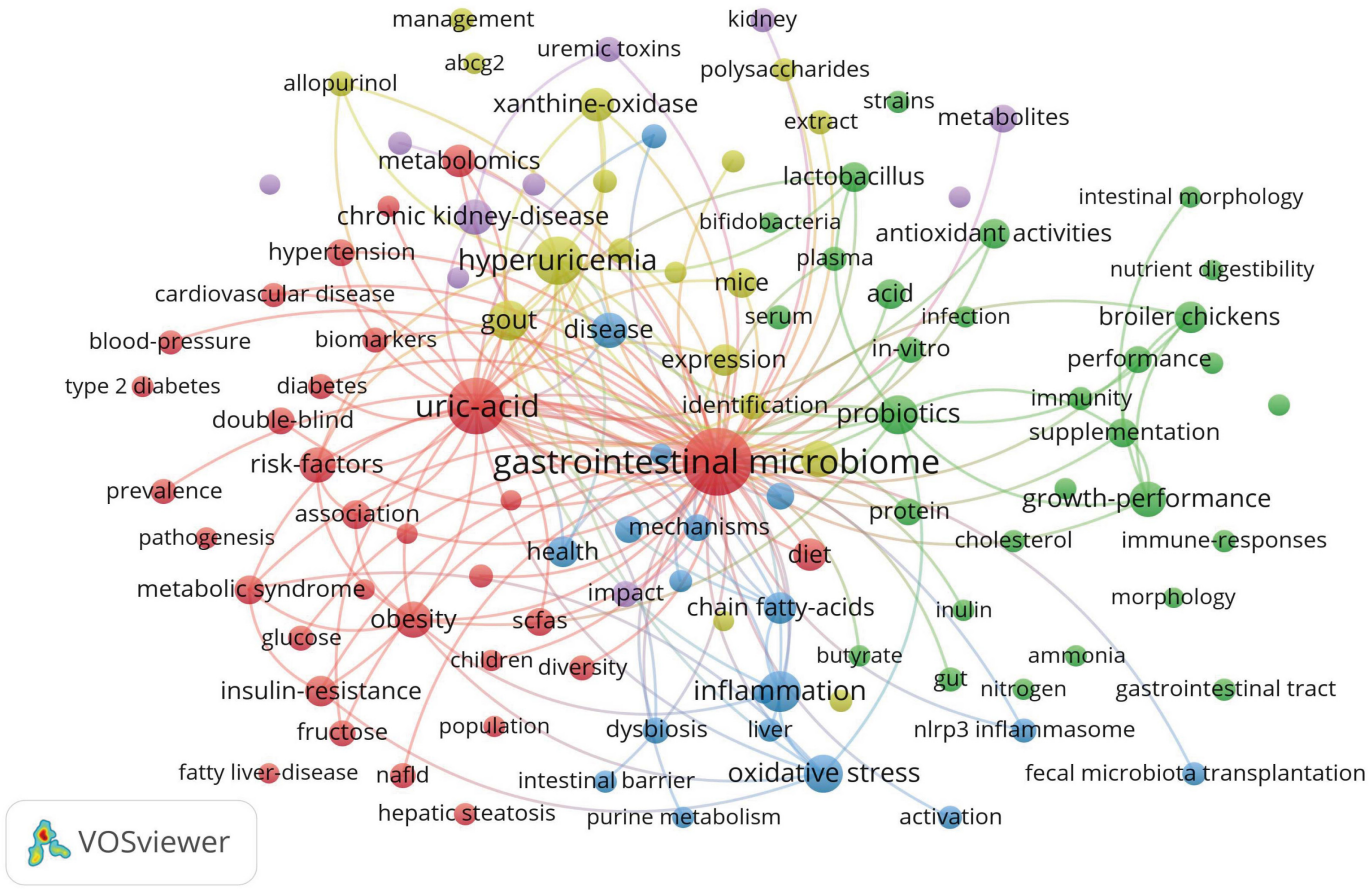
The co-occurrence network diagram of keywords related to gut microbiota and hyperuricemia.

**FIGURE 7:**
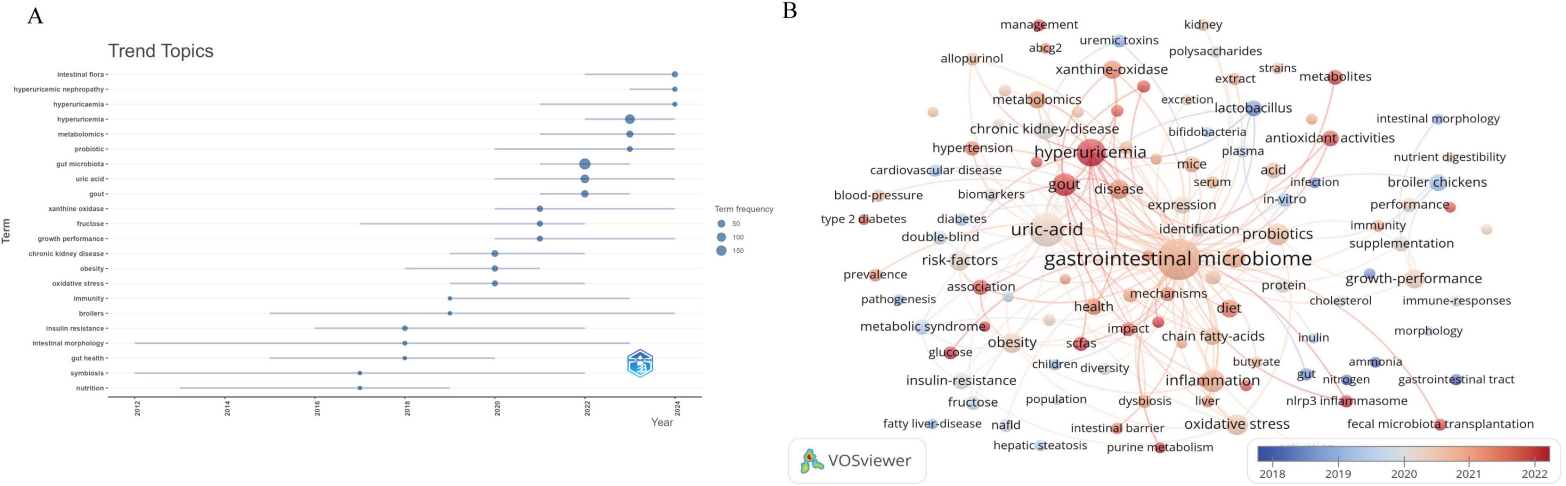
**(A)** The topic evolution map of hyperuricemia and gut flora. **(B)** Overlay visualization of hyperuricemia and gut flora.

High-frequency keywords can indicate evolving research frontiers within certain knowledge domains. Using VOSviewer, we identified 3,421 keywords, 109 of which appeared at least 10 times. Table 4 shows the top 20 high-frequency keywords. Among the keywords, “gastrointestinal microbiome” was the most frequently appeared (*n* = 500), followed by “uric-acid” (*n* = 280), “hyperuricemia” (*n* = 159), “inflammation” (*n* = 91), “gout” (*n* = 90), and “probiotics” (*n* = 80). Figure 6 depicts the co-occurrence of keywords that appeared more than 10 times. Each node’s size reflects how often it co-occurs, and the connections illustrate the relationships between these co-occurring keywords. Each link’s thickness reflects the frequency of co-occurrence between two keywords, with the same color representing a tighter cluster. Figure 6 results in the formation of five distinct clusters. Cluster 1, represented in red, concentrates on the pathogenesis within the field and its interactions with other diseases. This cluster encompasses 32 keywords, including gastrointestinal microbiome, UA, obesity, risk factors, diet, insulin resistance, and SCFA. Cluster 2, depicted in green, highlights the role of probiotics in regulating UA levels and their importance in improving gut health and growth performance in broiler chickens. This cluster comprises 30 keywords, such as probiotics, growth performance, broiler chickens, antioxidant activities, lactobacillus, immunity, inulin, and intestinal morphology. Cluster 3, shown in blue, addresses the molecular mechanisms pertinent to this field and the application of fecal microbiota transplantation. It includes 19 keywords, such as inflammation, oxidative stress, dysbiosis, NLRP3 inflammasome, and fecal microbiota transplantation. Cluster 4, illustrated in yellow, examines the mechanisms through which natural products modulate gut microbiota to ameliorate HUA and gout. This cluster includes 18 keywords such as hyperuricemia, gout, metabolism, xanthine oxidase, extract, polysaccharides, and pathway. Cluster 5 (purple cluster) focuses on the interactions between this field and kidney diseases, including 10 keywords such as chronic kidney disease, metabolites, kidney, impact, uremic toxins, and progression. To study the trend of theme changes in the field, we constructed a topic evolution map and overlay visualization using the Bibliometrix package in the R software environment and VOSviewer (Figure 7). Figure 7 displays the topic evolution and a high-frequency keyword overlay map, with colors indicating the average publication year. The analysis revealed that from 2012 to 2018, the field primarily focused on macro issues, including intestinal gut health status in HUA. From 2019 to 2024, the areas of “gut-kidney axis,” “SCFAs,” “antioxidant activities,” “fecal microbiota transplantation,” “probiotics,” “diet,” and “untargeted metabolomics” are notably emerging, as marked in yellow, focusing on intervention mechanisms between HUA and gut flora.

**TABLE 4:** The top 20 most common keywords.

Rank	Keyword	Occurrences	Rank	Keyword	Occurrences
1	Gastrointestinal microbiome	500	11	Growth-performance	58
2	Uric acid	280	12	Chronic kidney-disease	57
3	Hyperuricemia	159	13	Disease	57
4	Inflammation	91	14	Xanthine-oxidase	51
5	Gout	89	15	Diet	45
6	Probiotics	80	16	Metabolomics	43
7	Oxidative stress	73	17	Chain fatty-acids	42
8	Obesity	64	18	Expression	41
9	Metabolism	61	19	Broiler chickens	39
10	Risk-factors	60	20	Insulin-resistance	38

**FIGURE 8:**
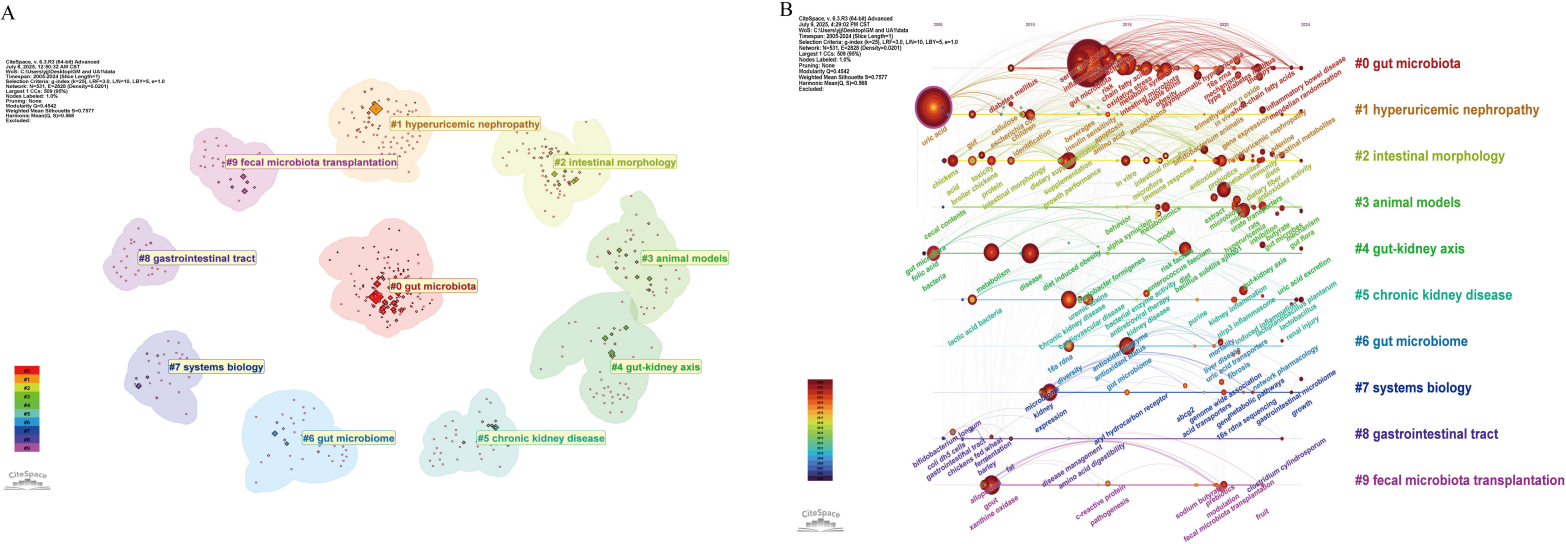
**(A)** Keyword clustering graph of hyperuricemia and gut flora. **(B)** Timeline visualization graph of keywords of hyperuricemia and gut flora.

Keyword clustering can visually present the topic distribution in the research field. In this study, the LSI clustering method of CiteSpace software was used to obtain a reasonable clustering graph (Figure 8, *Q* = 0.4542, *S* = 0.7577). A *Q* value exceeding 0.3 signifies a notable cluster structure. An *S* value above 0.5 suggests effective clustering, while values over 0.7 denote high reliability. As shown in Figure 8A, 10 major research clusters have been formed in this field. Different clusters contain distinct keywords and themes. Clusters with smaller label values encompass a broader range of keywords, indicating more diverse research content. The 10 clustering clusters are as follows: #0 gut microbiota, #1 hyperuricemic nephropathy, #2 intestinal morphology, #3 animal models, #4 gut-kidney axis, #5 chronic kidney disease, #6 gut microbiome, #7 systems biology, #8 gastrointestinal tract, and #9 fecal microbiota transplantation. Figure 8B is a timeline visualization map created by CiteSpace. The timeline visualization shows the first appearance time of each important keyword and the dynamic changes of research hotspots, reflecting the evolution of research topics. As shown in Figure 8B, from 2005 to 2012, researchers have begun to recognize that gut microbiota may be related to UA metabolism (e.g., gut microflora, microbiome, and UA). However, the research is still in its infancy. From 2013 to 2018, this field focused on the changes in the gut microbiota and related mechanisms of hyperuricemia, as well as the changes in the gut microbiota associated with metabolic diseases and high levels of UA (e.g., gut microbiota, double blind, oxidative stress, and metabolic syndrome). Since 2019, the research focus in this field has shifted to the microbial intervention effects and mechanisms of hyperuricemia (e.g., probiotics, prebiotics, extract, diets, and therapy). In addition, the gut-kidney axis, hyperuricemic nephropathy, SCFAs, butyrate, etc., have become the focus of research in recent years.

## 4 Discussion

### 4.1 Basic information

A total of 736 papers on HUA and gut flora were included in this study. The publication volume in this field can be divided into three phases: a low activity period (2005–2012), a slow growth period (2013–2019), and an active period (2020–2024). This increasing trend reflects the growing interest and contributions of researchers in the field. The advancement and use of new technologies like metagenomics and high-throughput sequencing undoubtedly aid the growth in this discipline (Qin et al., 2010; Weinstock, 2012). Several nations or institutions have repeatedly carried out gut microbiota-related initiatives and achieved ground-breaking results, which have also offered direction and established the groundwork for the research into the connection between gut flora and HUA. For instance, the European Commission launched the Human Gut Metagenome Project in 2008, and the National Institutes of Health in the United States published the Human Microbiome Project (HMP) in 2007 (Turnbaugh et al., 2007; The Human Microbiome Project Consortium, 2012).

#### 4.1.1 Countries/regions and institutions

China ranks first in the number of publications in this field, followed by the United States. The publication output of these two countries far exceeds that of others, indicating the significant interest of researchers in both countries and their substantial investment in research related to gut microbiota and HUA. Among the top 10 institutions in terms of publications, the top institution in terms of both publications and citations is UCSD in the United States, and the remaining 9 institutions are from China. UCSD has close collaboration with leading institutions such as Qingdao University and the Chinese Academy of Sciences. Professor Changgui Li from the Department of Endocrinology and Metabolism at the Affiliated Hospital of Qingdao University, Professor Huiyong Yin from the Chinese Academy of Sciences, and expert Robert Terkeltaub from UCSD worked together to discover differentially abundant metabolites and pathways underlying infrequent gout flares (InGFs) and frequent gout flares (FrGFs) through metabolomics and to establish a predictive model via machine learning (ML) algorithms (Wang M. et al., 2023). Notably, Robert Terkeltaub contributed to the American College of Rheumatology’s gout management guidelines (Khanna et al., 2012). Additionally, Professor Changgui Li serves as co-chair of the Asia–Pacific Gout Consortium (APGC)^2^ and plays a leading role in the field of gout and HUA, potentially laying the groundwork for collaboration among various organizations.

#### 4.1.2 Authors

Analysis of authors and co-cited authors demonstrates that Sanjay K. Nigam is an influential writer who has made significant contributions to the fields of HUA and the intestinal microbiota. A 2015 review by Sanjay K. Nigam, published in Physiological Reviews, stands out as a representative work. This review underscores the essential functions of organic anion transporter 1 (OAT1) and OAT3 in the metabolism and processing of gut microbiome metabolites (Nigam et al., 2015). These transporters are predominantly expressed in the proximal tubule cells of the kidney, where they facilitate the transport of organic anions, such as UA, from the bloodstream into cells, subsequently expelling them through the cell’s apical membrane into the urine. OAT1 and OAT3 are particularly critical in the transmembrane transport of UA, a process they facilitate by exchanging dihydroxyacetate within the cell (Otani et al., 2017). In 2019, Sanjay K. Nigam’s team constructed a co-expression network of the gut-liver-kidney (GLK) axis, revealing interactions between transport proteins (e.g., OAT1, OAT3, and URAT1) and metabolizing enzymes (e.g., CYP4A11 and UGT2B4) associated with UA metabolism, emphasizing the role of these genes in regulating UA levels and intestinal flora metabolite transport (Rosenthal et al., 2019). In 2020 and 2022, it was noted that OAT1 and OAT3 are involved in UA reabsorption and excretion in renal proximal tubules and interact with gut flora metabolites (e.g., indoleacetic acid and 4-hydroxyphenylacetic acid) to affect UA levels, which in turn affects the progression of CKD (Engelhart et al., 2020; Granados et al., 2022; Jamshidi and Nigam, 2022). In 2023, it was found that gut microbiota metabolites, including tryptophan derivatives, could activate the host’s OATs and aryl hydrocarbon receptor (AHR), impacting UA secretion and excretion, thus creating a remote sensing and signaling mechanism between UA metabolism and intestinal flora, which is particularly important in chronic kidney disease (Nigam and Granados, 2023). In addition, loss of function of transporter proteins such as OAT1 leads to changes in the composition and function of the intestinal flora, thereby affecting UA metabolism (Ermakov et al., 2023). The research of Professors Sanjay K. Nigam et al. revealed the core bridging role of OAT1/OAT3 in the interaction between UA metabolism and intestinal microbiota, constructed the theoretical framework of the GLK axis, and laid a revolutionary theoretical foundation for the mechanism research, prevention, and treatment of hyperuricemia and chronic kidney disease.

#### 4.1.3 Journals

A total of 736 documents appeared in 369 different journals, with major contributions from respected sources like Nutrients, Frontiers in Microbiology, and Food & Function. Interestingly, the Nutrients stood out as a primary focus, with a significant number of published studies and citations. This recognition confirms the Nutrients’ position as a significant outlet for sharing research findings in the field of gut microbiota and HUA and highlights the publication’s importance in this sector.

#### 4.1.4 References

References with the strongest citation bursts analysis reveal the most influential references in the field. The citation boom period in this field is relatively concentrated, mainly occurring between 2017 and 2022. The citation research content that emerged at this stage was quite rich, including the description of the microbiota of clinical patients and microecological intervention strategies (including probiotics for lowering UA, dietary fiber for promoting the generation of SCFAs, tuna oligopeptides for repairing the intestinal barrier, etc.) (Guo et al., 2016; Shao et al., 2017; García-Arroyo et al., 2018; Han et al., 2020). Published in 2016 in Scientific Reports, the paper “Intestinal Microbiota Distinguish Gout Patients from Healthy Humans” experienced the strongest citation burst (strength = 17.67) between 2017 and 2021. This is a clinical cohort study, which revealed significant differences in the intestinal microflora between gout patients and healthy individuals. *Bacteroides xylanisolvens* and *Bacteroides caccae* were more abundant in gout patients, whereas *Bifidobacterium pseudocatenulatum* and *Faecalibacterium prausnitzii* were less abundant (Guo et al., 2016). The second strongest citation burst (strength = 13.27) article, titled “Combined Signature of the Fecal Microbiome and Metabolome in Patients with Gout,” was published by Shao et al. (2017) in the journal Frontiers in Microbiology. This study examined the fecal microbiome signatures and revealed an increase in pathogens, including *Erysipelatoclostridium*, *Rhodococcus*, *Anaerolineaceae*, and *Bacteroides*. The metabolome signatures included altered metabolites that may play a role in inflammatory responses, purine metabolism, and UA excretion (Shao et al., 2017). The third most intense citation burst (strength = 8.8) is an article titled “Alterations of the Gut Microbiome Associated With the Treatment of Hyperuricaemia in Male Rats,” published by Yu et al. (2018) in the journal Frontiers in Microbiology. The study investigates the effects of allopurinol and benzbromarone on the gut microbiota of male rats with hyperuricaemia, revealing specific alterations in bacterial genera and metabolic pathways associated with nucleotide and lipid metabolism (Yu et al., 2018). In addition, several papers that are still in the explosive period (2022–2024) indicate that the functional differences of gout subtypes in the microbiota and the deepening of probiotic mechanisms in this field have been studied (Méndez-Salazar et al., 2021; Ni et al., 2021).

### 4.2 Research hotspots and trends

The analysis of keywords can highlight the essential topics, focal points, and trends in a research domain, aiding researchers in grasping the knowledge framework and potential future directions of the field. By analyzing the high-frequency keywords, co-occurrence of keywords, keyword clustering, and timeline visualization maps in the research field of intestinal flora in hyperuricemia, the primary focuses of current studies can be identified.

#### 4.2.1 Features of the gut microbiota in individuals with HUA

Through keyword co-occurrence and timeline visualization graphs, a large number of microbiota names, such as “gut microbiota,” “Bifidobacteria,” and “Escherichia coli,” were discovered, indicating that various types of microbiota have always been the focus of attention in this field. Multiple studies have shown that compared with healthy individuals, there are significant differences in the diversity and composition of the gut microbiota in patients with HUA (Table 5 and Supplementary Table 1). These differences are not only reflected in the “rise and fall” of specific bacterial genera, but also reflect the dual impact of the imbalance in the interaction between the microbiota and the host on metabolism and inflammation. Firstly, the enrichment of SCFAs-producing genera such as *Alistipes*, *Faecalibacterium*, and *Roseburia* has been repeatedly reported (Lin et al., 2021; Yuan et al., 2022). These bacteria may constitute a compensatory mechanism by which the body attempts to alleviate hyperuric acid-related metabolic disorders by maintaining the integrity of the intestinal barrier and inhibiting inflammatory responses. Secondly, multiple studies have reported that serum UA levels are positively correlated with the abundance of *Bacteroides* in patients with HUA/gout (Guo et al., 2016; Shao et al., 2017; Méndez-Salazar et al., 2021). Although research reports have found that *Bacteroides* are one of the main enterotypes in the healthy population of South Korea, and 5-hydroxyisourate hydrolase, involved in the conversion of UA to allantoin, is enriched in this type of intestinal type (Lim et al., 2014). However, most studies have found that *Bacteroides* are involved in pro-inflammatory effects under disease conditions. For instance, B. caccae have been identified as biomarkers of inflammatory bowel disease (IBD) (Wei et al., 2001). Additionally, an increased presence of Bacteroides spp. has been linked to various autoimmune diseases, including systemic lupus erythematosus (Hevia et al., 2014), rheumatoid arthritis (Zhang et al., 2015), and type 1 diabetes (Davis-Richardson and Triplett, 2015). It is suggested that the amplification of Bacteroides may simultaneously trigger chronic inflammation. Third, the general reduction of probiotics such as Bifidobacteria (Guo et al., 2016; Lin et al., 2021; Méndez-Salazar et al., 2021; Yang et al., 2021; Ul-Haq et al., 2022). Bifidobacteria are renowned for their probiotic properties and anti-inflammatory effects, and their depletion may contribute to the overall dysbiosis and elevated inflammatory levels in HUA patients (Ruiz et al., 2017). In addition, the imbalance of opportunistic pathogenic bacteria (such as Porphyromonas and Enterobacteriaceae) and SCFA-producing beneficial bacteria (such as *Clostridium* and *Ruminococcus*) further amplifies the risk of inflammation. It is worth noting that the abundance changes of *Prevotella* show contradictory results in different studies (Ul-Haq et al., 2022; Martínez-Nava et al., 2023). This discrepancy highlights the influence of confounding factors such as disease stage, diet, and testing methods on the dynamics of the microbiota (Wu et al., 2011). In conclusion, the intestinal microbiota of patients with HUA shows a pattern of “pro-inflammatory-anti-inflammatory” bacterial growth and decline. This imbalance pattern may either be the result of metabolic disorders or exacerbate hyperuricemia and inflammation through gut-axis feedback. In the future, it is necessary to combine longitudinal cohorts and standardized analyses to clarify the causal roles and individual heterogeneity of the changes in the microbiota. Meanwhile, the specific mechanism of action of *Bacteroides* under different physiological and pathological conditions should be further explored to provide more comprehensive theoretical support for the prevention and treatment of hyperuricemia and related diseases.

**TABLE 5:** Changes in gut flora in patients with hyperuricemia and gout.

Flora	Variations in abundance	Flora	Variations in abundance
*Bacteroides*	↑	*Clostridium*	↓
*Porphyromonadaceae*	↑	*Bifidobacterium*	↓
*Anaerolineaceae*	↑	*Ruminococcaceae*	↓
*Fusobacteria*	↑	*Coprococcus*	↓
*Prevotella*	↑	*Prevotellaceae*	↓
*Faecalibacterium*	↑	*Butyricicoccus*	↓
*Enterobacteriaceae*	↑	*Oscillibacter*	↓

#### 4.2.2 Mechanisms by which probiotics regulate uric acid levels

Through high-frequency keywords, keyword clustering, and citation burst analysis, we found that “probiotics,” “prebiotics,” and “therapy” are important research topics in the field of gut microbiota in hyperuricemia. The mechanism of probiotics in treating HUA includes altering the composition of the intestinal microbiota. Probiotics regulate the composition of the gut microbiota by increasing the abundance of beneficial bacteria (such as *Bifidobacteria*, *Prevotella*, *Firmicutes*, and SCFA-producing bacteria) while reducing pathogenic or pro-inflammatory bacteria (such as *Bacteroidetes* and *Enterococcus*). For instance, researchers have shown that *Lactobacillus plantarum LLY-606* and *L. plantarum TCI227* enhance the production of SCFAs and decrease the abundance of harmful bacteria such as *Escherichia/Shigella* (Chien et al., 2022; Shi et al., 2023). This modulation of the gut microbiota is considered a key mechanism underlying the therapeutic efficacy of probiotics. Secondly, it inhibits the activity of XOD. XOD is a pivotal enzyme in the biosynthesis of UA. Several probiotic strains (including *Lactobacillus rhamnosus Fmb14*, *L. plantarum Q7*, and *Lactobacillus DM9218*) have been shown to inhibit XOD activity in the liver, subsequently reducing UA concentrations (Wang et al., 2019; Ni et al., 2021; Cao et al., 2022; Li et al., 2022; Zhao et al., 2022; Shi et al., 2023). Additionally, probiotics can modulate the expression of UA transporter proteins, such as ABCG2, GLUT9, and URAT1 (Zhao et al., 2022; Wang Z. et al., 2023). These observations underscore the potential of probiotics to exert a direct influence on UA metabolism. Furthermore, numerous probiotics demonstrate notable anti-inflammatory properties by diminishing the expression of pro-inflammatory cytokines, including IL-1β, TNF-α, and IL-6. For instance, *L. rhamnosus Fmb14* and *L. plantarum LLY-606* have been shown to downregulate IL-1β and TNF-α levels, thereby mitigating inflammation associated with HUA (Li et al., 2022; Shi et al., 2023). Supplementary Table 2 details the specific mechanisms.

In addition, keyword co-occurrence reveals that the high-frequency keywords “growth performance,” “broiler chickens,” and “probiotics” are linked to each other. It is indicated that adding probiotics to regulate the UA level in broilers and improve their growth performance and meat quality is also a research hotspot on this topic. For instance, studies have shown that *Lactobacillus farciminis CNMA67-4R*, *Clostridium butyricum CBM 588*, multi-strain probiotics, and symbiotics (*Bacillus subtilis*, *inulin*, and *Saccharomyces cerevisiae*) significantly reduced the UA levels in the feces of broiler chickens, which is the primary substrate for ammonia production. Moreover, *L. farciminis CNMA67-4R* and the symbiotics decreased the urine nitrogen ratio in the feces, thereby leading to a reduction in ammonia emissions from the chickens (Such et al., 2021; Such et al., 2023). The reduction of ammonia emissions is of considerable importance for environmental protection and animal welfare, and it can also enhance the growth performance and immune function of broiler chickens. The timeline visualization map shows that “fecal microbiota transplantation,” “fermentation,” and “extract” are the key research topics in recent years. It indicates that, in addition to probiotics, other microbiota-based treatments [such as microbiota transplantation and microbial fermentation extracts (MFEs)] have received extensive attention. Liu et al. (2020) found that FMT effectively modulated the gut microbiota in rats with high-purine-induced HUA, leading to improvements in metabolic parameters. Furthermore, in patients with gout, washing microbiota transplantation decreases serum UA levels, is related to a reduction in both the frequency and duration of acute gout flares, lowers diamine oxidase and endotoxin levels, and helps improve their impaired intestinal barrier function (Xie et al., 2022). Treatment with *Lactobacillus acidophilus* fermented dandelion (LAFD) has been shown to restore imbalances in the gut microbial ecosystem and reverse alterations in *Bacteroidetes/Firmicutes*, *Muribaculaceae*, and *Lachnospiraceae* in HUA mice (Ma et al., 2023). In addition, research indicates that certain MFEs did not exhibit systemic toxicity even at high doses, suggesting a favorable safety and efficacy profile for the treatment and prevention of HUA (Chen et al., 2017). In conclusion, the technique of bacterial colony transplantation and its associated microbial fermentation products hold promise for the treatment of HUA.

However, in practical applications, probiotics face multiple challenges in the management of HUA. Firstly, ensuring a high viable bacterial count and stability of probiotics during processing, storage, transportation, and after passing through harsh gastrointestinal environments such as gastric acid and bile is crucial. Although encapsulation technology can enhance targeted delivery and viability, its effectiveness and repeatability still need to be optimized (Broeckx et al., 2016; Cassani et al., 2020; Sun et al., 2023). Secondly, significant individual differences in therapeutic effects exist, influenced by the composition of the host’s intestinal flora, genetic background, and lifestyle factors such as diet and medication (Sun et al., 2024; Wang Q. et al., 2024). This highlights the necessity of personalized strain and dosage selection. Finally, the current regulatory and standardization framework is still not perfect. There is a lack of unified standards for strain identification, evaluation, and quality control. It is urgent to implement stricter quality control procedures (such as ensuring the consistency between the actual content of the product and the label statement and preventing contamination) and adopt genomic assessment methods to verify the safety and accuracy of strains, to enhance product quality, safety, and consumer trust (Salvetti et al., 2016; Kolaček et al., 2017).

#### 4.2.3 Microecological interventions

Keyword co-occurrence and timeline visualization analysis reveal that “polysaccharides,” “diet,” and “dietary fiber” are important research topics in this field, indicating that both traditional Chinese medicine (TCM) and dietary patterns regard the intestinal flora as the hub target for regulating HUA. They exert their effects through a two-way intervention strategy: on the one hand, they inhibit “dangerous bacteria” such as *Bacteroidetes* and *Enterobacteriaceae* that produce endotoxins and promote inflammation; on the other hand, they increase beneficial bacterial species such as Lactobacillus, Bifidobacterium, and Roseburia that produce SCFAs and strengthen the gut-kidney axis. Ultimately, they jointly reduce the UA load, decrease oxidative stress and inflammation, and achieve multi-layered benefits from the structure of the microbiota to the host’s metabolic-immune-kidney function.

The active ingredients of TCM and the intestinal flora synergistically intervene in UA homeostasis through a “bidirectional metabolism-regulation” model. Some bioactive ingredients in TCM are not effectively absorbed in the digestive tract, causing low bioavailability (Gong et al., 2020). On the one hand, TCM is first enzymatically hydrolyzed by the microbiota to generate more easily absorbed active metabolites, such as baicalin being converted into baicalein before entering the bloodstream (Noh et al., 2016), and the host metabolism is indirectly regulated through SCFAs and other microbiota products (Wang et al., 2017); on the other hand, active ingredients reshape the structure of the microbiota. Tea polyphenols, berberine, etc., can inhibit harmful bacteria, promote the growth of beneficial bacteria, improve microecology, and UA metabolism (Yan et al., 2020; Chen Q. et al., 2023; Wang L. et al., 2023). This bidirectional network provides new ideas for explaining the mechanism of action of TCM and developing microbiogenic therapies. However, most of the existing evidence remains at the animal model level, and the causal chain has not yet been fully verified in the human body. Among polyphenols, resveratrol (from *Polygonum cuspidatum*) was shown to increase *Lactobacillus* spp. and SCFA-producing bacteria (e.g., *Clostridium* and *Bifidobacterium*), while reducing *Bacteroides* and pro-inflammatory cytokines (IL-6, IL-1β, and TNF-α) (Zhou Y. et al., 2024). Chlorogenic acid (from *Lonicera japonica*) was observed to inhibit TMAO-synthesizing bacteria (*Faecalibaculum* and *Blautia*) and activate the PI3K/AKT/mTOR pathway, reducing renal fibrosis and oxidative stress (Zhou et al., 2022). Furthermore, ferulic acid (from *Ligusticum chuanxiong*) remodeled gut microbiota in HUA rats, while inhibiting the TLR4/NF-κB pathway to reduce UA absorption (Zhang N. et al., 2023). Among flavonoids, quercetin (from *Taxillus chinensis*) reduced hepatic XOD activity and enhanced purine degradation in HUA mice, with *Lactobacillus aviarius* implicated in UA metabolism modulation (Li D. et al., 2023). Myricetin-Nobiletin Hybrid (from waxberry and *Citrus reticulata*) regulated glycerophospholipid metabolism and increased *norank_f_Muribaculaceae* abundance in HUA mice, ameliorating renal damage (Li Y. et al., 2023). Saffron flavonoid extract (from *Crocus sativus*) reversed HUA-induced dysbiosis by enriching beneficial genera (*Roseburia* and *Clostridium* sp.) and suppressing pathogens (*Alloprevotella* and *Parabacteroides*) in rats, while enhancing antioxidant activity (Chen et al., 2022). Regarding alkaloids, berberine (from *Coptis chinensis*) upregulated colonic ABCG2 and reduced *Bacteroidetes* in HUA rats, enriching *Lactobacillus* and promoting UA excretion (Chen Q. et al., 2023). The classification of active ingredients in TCM, as well as their mechanisms of action and targeted bacterial communities, is shown in Supplementary Table 3.

**FIGURE 9:**
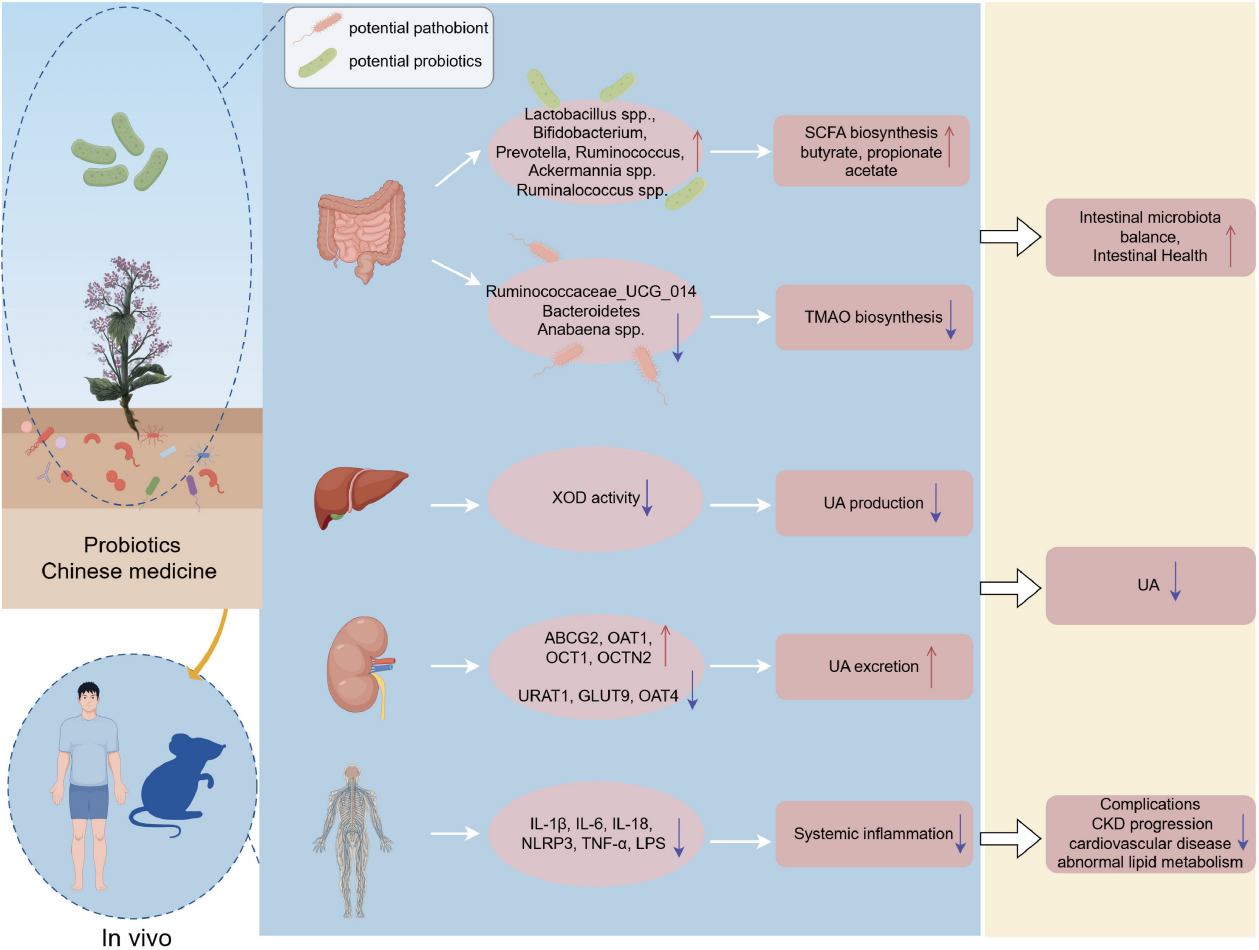
Mechanism of probiotics and TCM in regulating gut microbiota to alleviate hyperuricemia. Probiotics and TCM components influence gut microbiota by promoting beneficial bacteria and inhibiting harmful ones, while also modulating metabolic pathways to lower UA levels, reduce inflammation, and enhance gut health. Created by FigDraw.

In addition, diuretic and turbidium-reducing herbs have also demonstrated consistent effects in animal models, such as enhancing probiotics, reducing pathogenic bacteria, and lowering UA. Kidney tea has been observed to significantly enhance the abundance of *Roseburia* and *Enterorhabdus*, while reducing the abundance of *Ileibacterium* and *UBA1819* in HUA model mice (Chen Y. et al., 2023). In addition, Chicory promotes probiotic growth and pathogen reduction to aid UA excretion in HUA quail (Bian et al., 2020). Additionally, *Camellia sinensis* increases the abundance of *Ruminococcus* and *Lactobacillus*, decreases the abundance of *Bacteroides* and *E. coli*, and regulates UA metabolism in HUA mice (Wu et al., 2022). Although preclinical studies based on rodent models suggest that TCM and its components may intervene in HUA by regulating the intestinal flora and UA metabolism, there is still significant uncertainty regarding its clinical transformation. The key limitation lies in the lack of high-quality clinical trials to verify the mechanism of action mediated by microorganisms. It is difficult to establish a causal relationship in multi-component compound prescriptions where specific components drive microbial changes and mechanically reduce UA. The methodological heterogeneity existing in preclinical studies (such as differences in HUA induction regimens, administration doses/dosage forms, and microbiota analysis techniques) restricts the direct comparability and universality of the results. Furthermore, the long-term sustainability impact of significant baseline microbiota differences among individuals on intervention responses and induced changes urgently needs in-depth exploration. Future research should enhance high-quality clinical trials, adopt uniform standards, systematically evaluate the impact of TCM on HUA, and take into account individual differences in microbiota to formulate personalized treatment plans. Figure 9 demonstrates the mechanisms by which probiotics and TCM mitigate HUA through modulation of the gut microbiota.

Different dietary patterns significantly influence the composition and function of gut microbiota, which in turn affects the management of HUA. A study has demonstrated that dietary patterns characterized by high intake of animal protein, fat, and alcohol are positively correlated with the prevalence of HUA, whereas diets abundant in fruits, vegetables, legumes, and grains are inversely associated with HUA prevalence (Teng et al., 2015). The key components of these dietary patterns regulate UA levels through specific interactions with gut microbiota and their metabolic outputs. For example, Western dietary patterns, characterized by elevated intake of saturated fats and animal proteins, induce dysbiosis of the intestinal flora (Huang et al., 2013). The microbiota characteristics of animal models induced by long-term high-fat and high-fructose diets also support this view (Zhang N. et al., 2023). This dysbiosis increases intestinal permeability and stimulates the release of pro-inflammatory mediators (e.g., LPS, TNF-α, and IL-1β) and diminishes the production of SCFAs, particularly butyrate, which has anti-inflammatory properties and may enhance renal UA excretion (Chu et al., 2021; Li et al., 2021; Xu et al., 2021). In addition, researchers have found that SCFAs enhance the excretion of intestinal UA by activating peroxisome proliferator-activated receptor γ (PPARγ), which binds to the ABCG2 promoter (Adeyanju et al., 2021; Li M. et al., 2023). Conversely, the Mediterranean dietary (e.g., fruits, vegetables, nuts, whole grains, and beans) pattern, abundant in vitamins, minerals, polyphenols, dietary fiber, and monounsaturated fatty acids (MUFAs), fosters the proliferation of *Bifidobacteria* and *Lactobacillus*, and improve gut health by increasing the production of SCFAs while mitigating cells inflammatory responses lowering concentrations of TMAO, thus exerting a favorable effect on UA reduction (Chrysohoou et al., 2011; Chatzipavlou et al., 2014; Cariello et al., 2020; Soldán et al., 2024). Research has demonstrated that in hyperuricemia nephropathy rats, there was a significant increase in *Blautia*, *Enterococcus*, and *Faecalibaculum* associated with TMAO production. TMAO activates PI3K/AKT/mTOR signaling pathway, induces local inflammatory reaction in the kidney, aggravates kidney fibrosis, destabilizes UA transport proteins, and diminishes the kidney’s UA excretion capacity (Zhou et al., 2022). In addition, The Dietary Approaches to Stop Hypertension (DASH) diet, which emphasizes plant-based foods rich in whole grains, fruits, vegetables, and low-fat dairy products, has been shown to significantly enhance the abundance of beneficial gut microbiota such as *Bifidobacterium* and *Lactobacillus*, while reducing the prevalence of harmful bacteria, including LPS-producing *Enterobacteriaceae*. These beneficial microbial populations contribute to the reduction of UA levels through the production of SCFAs and the inhibition of XOD activity (Martínez et al., 2010; Gong et al., 2018; Ashaolu, 2020; Aslam et al., 2020). These findings underscore the significant role of dietary patterns in managing HUA by modulating the composition and function of the gut microbiome. Furthermore, selecting diets rich in dietary fiber and polyphenols may aid in lowering UA levels and alleviating symptoms in individuals with HUA.

#### 4.2.4 Quadruple-axis synergistic regulation of uric acid homeostasis by gut microbiota

Timeline visualization analysis revealed that “liver,” “kidney,” “gut-kidney axis,” etc. have also been research hotspots in recent years. It has attracted much attention in recent years that the gut microbiota, through a multi-dimensional network composed of four axes including gut-kidney, gut-liver, gut-joint, and gut-brain, collaboratively regulates UA homeostasis in distant organs. On the gut-kidney axis, probiotics (such as *L. rhamnosus*) directly upregulate colonic UA transporters (such as ABCG2), accelerating UA excretion to reduce blood UA (Wang H. et al., 2024). Its metabolites, SCFAs, indirectly maintain UA balance by strengthening the intestinal barrier and inhibiting the TLR4/NLRP3 inflammatory pathway (Bi et al., 2024; Wang Q. et al., 2024; Lv et al., 2025). In the gut-liver axis, specific strains (*Lactobacillus reuteri* and *Lactobacillus johnsonii*) also promote UA excretion by enhancing barrier function and transporter protein expression, while further reducing blood UA by utilizing purine metabolism and using UA as a carbon source (Kasahara et al., 2023; Hussain et al., 2024; Han et al., 2025). The combination of probiotics with ursolic acid and oleanolic acid can reshape the structure of the microbiota, synergistically inhibit liver inflammatory pathways, and optimize UA metabolism (Ma et al., 2020). On the gut-joint axis, the barrier disruption mediated by TLR4/NLRP3 inflammation hinders UA excretion, while 7-ketocholic acid promotes epithelial repair by inhibiting FXR (Li et al., 2024; Wang Q. et al., 2024). Probiotics enrich *Lactobacillus* and *Faecalibacterium* through tryptophan metabolism, enhance the UA transport function of the colon, and indirectly reduce the risk of hyperuricemia. The gut-brain axis enhances the barrier through strains such as *L. reuteri*, upregulates the production of ABCG2 and SCFAs, transmits signals to the central nervous system via the vagus nerve, and regulates the synthesis and excretion of UA (Cryan et al., 2019; Wang et al., 2020; Hussain et al., 2024). In conclusion, the collaborative network of microbiota, metabolites, transport proteins, and distal organs provides a new microbiogenic intervention strategy for hyperuricemia.

### 4.3 Strengths and limitations

As the first thorough systematic bibliometric analysis of the gut microbiota and HUA, our work offers a wealth of insights and directions for academic investigators and clinical professionals alike. However, this study has the following limitations. Firstly, the research data were sourced solely from the WOS and PubMed databases, possibly resulting in incomplete data and outcomes. Secondly, as the bibliometric analysis tool of this study relies primarily on English literature, it may neglect non-English literature, potentially impacting a comprehensive understanding of global research activities. Thirdly, existing research shows that differences influence variations in gut microbiota characteristics among HUA patients in study subjects, sample sizes, detection methods, geographic locations, and dietary habits. The gut microbiota composition in animal models of HUA varies, likely due to differences in animal types and preparation methods. These variations may lead to inadequacies in concluding. Finally, bibliometric analysis needs to keep pace with actual research activities, and the updating of citation data takes time, potentially resulting in inadequate responsiveness to emerging research areas or hot topics.

## 5 Conclusion

In this extensive bibliometric analysis, we employed CiteSpace, VOSviewer, and Bibliometrix to systematically analyze a substantial corpus of research concerning HUA and the gut microbiota. Overall, our research has revealed four important research hotspots in this field, including microbiota characteristics, probiotic therapy, microecological intervention, and the gut-distal target organ axis. The focus of emerging hotspots is on dietary supplementation, microbiota transplantation treatment strategies, and extensive research on the organ axes discussed above. Moreover, although the modulation of the gut microbiota to improve HUA and its related diseases shows great potential, there are still some shortcomings in understanding broader mechanisms, long-term effects, and clinical applications. Future research should consider conducting large-scale, long-term clinical trials to assess the efficacy and safety of different microbiome therapies and utilize multi-omics technologies (including but not limited to proteomics and transcriptomics) to explore a wide range of molecular mechanisms to address these gaps.

## Data Availability

The original contributions presented in this study are included in this article/Supplementary material, further inquiries can be directed to the corresponding authors.

## Footnotes

1
https://www.bibliometrix.org/

2
https://ggwsxy.qdu.edu.cn/info/1171/3404.htm
