# Supplementary material for: Hyperuricemia and the gut microbiota: current research hotspots and future trends
Source: Front Microbiol. 2025 Aug 14;16:1620561. doi: 10.3389/fmicb.2025.1620561 (PMC12391128; doi:10.3389/fmicb.2025.1620561)
Supplement: Supplementary file 1 [file Supplementary_file_1.docx]

**Hyperuricemia and the Gut Microbiota: Current Research Hotspots and Future Trends.**

**Appendix 1**

**Hyperuricemia and the gut microbiota term**

((hyperuricemia) or (hyperuricemic) or (hyperuricemia) or (hyperuricaemia) or (gout) or (uric acid) or (acid, uric) or (2,6,8-Trihydroxypurine) or (trioxopurine) or (potassium urate) or (urate, potassium) or (urate) or (ammonium acid urate) or (acid urate, ammonium) or (urate, ammonium acid) or (sodium urate monohydrate) or (monohydrate, sodium urate) or (urate monohydrate, sodium) or (monohydrate, monosodium urate) or (urate monohydrate, monosodium) or (sodium acid urate monohydrate) or (sodium urate) or (urate, sodium) or (monosodium urate) or (urate, monosodium) or (sodium acid urate) or (acid urate, sodium) or (urate, sodium acid)) and ((gastrointestinal microbiomes) or (microbiome, gastrointestinal) or (gut microbiome) or (gut microbiomes) or (microbiome, gut) or (gut microbiota) or (gut microbiotas) or (microbiota, gut) or (gastrointestinal flora) or (flora, gastrointestinal) or (gut flora) or (flora, gut) or (gastrointestinal microbiota) or (gastrointestinal microbiotas) or (microbiota, gastrointestinal) or (gastrointestinal microbial community) or (gastrointestinal microbial communities) or (microbial community, gastrointestinal) or (gastrointestinal microflora) or (microflora, gastrointestinal) or (gastric microbiome) or (microbiome, gastric) or (gastric microbiomes) or (intestinal microbiome) or (intestinal microbiomes) or (microbiome, intestinal) or (intestinal microbiota) or (intestinal microbiotas) or (intestinal microflora) or (microflora, intestinal) or (intestinal flora) or (flora, intestinal) or (enteric bacteria) or (bacteria, enteric) or (fecal microbiome) or (intestinal bacteria) or (gut bacteria) or (gastrointestinal bacteria) or (gut microflora) or (fecal microflora))
