# Supplementary material for: Hyperuricemia and the gut microbiota: current research hotspots and future trends
Source: Front Microbiol. 2025 Aug 14;16:1620561. doi: 10.3389/fmicb.2025.1620561 (PMC12391128; doi:10.3389/fmicb.2025.1620561)
Supplement: Supplementary file 2 [file Supplementary_file_2.docx]

Hyperuricemia and the Gut Microbiota: Current Research Hotspots and Future Trends.

Appendix 2

Supplementary Figure 1. The associations map among countries, affiliations, and authors…………………………………………………………………………………………………………………………………2

Supplementary Figure 2. Dual-map overlay of journals…………………………………...…………………………………………………………………………………………………………………………………3

Supplementary Table 1. Gut microbiota composition in patients with HUA and gout…………………………………………………………………………………………………………………………………….…4

**Supplementary Table 2.** Mechanisms of Probiotic Therapy for HUA.…………………...………………………...…………………………………………………………………………………………………………5

**Supplementary Table 3.** Active ingredients of TCM targeted gut microbiota Therapy for HUA.…………………...………………………...………………………………………………………………………………6


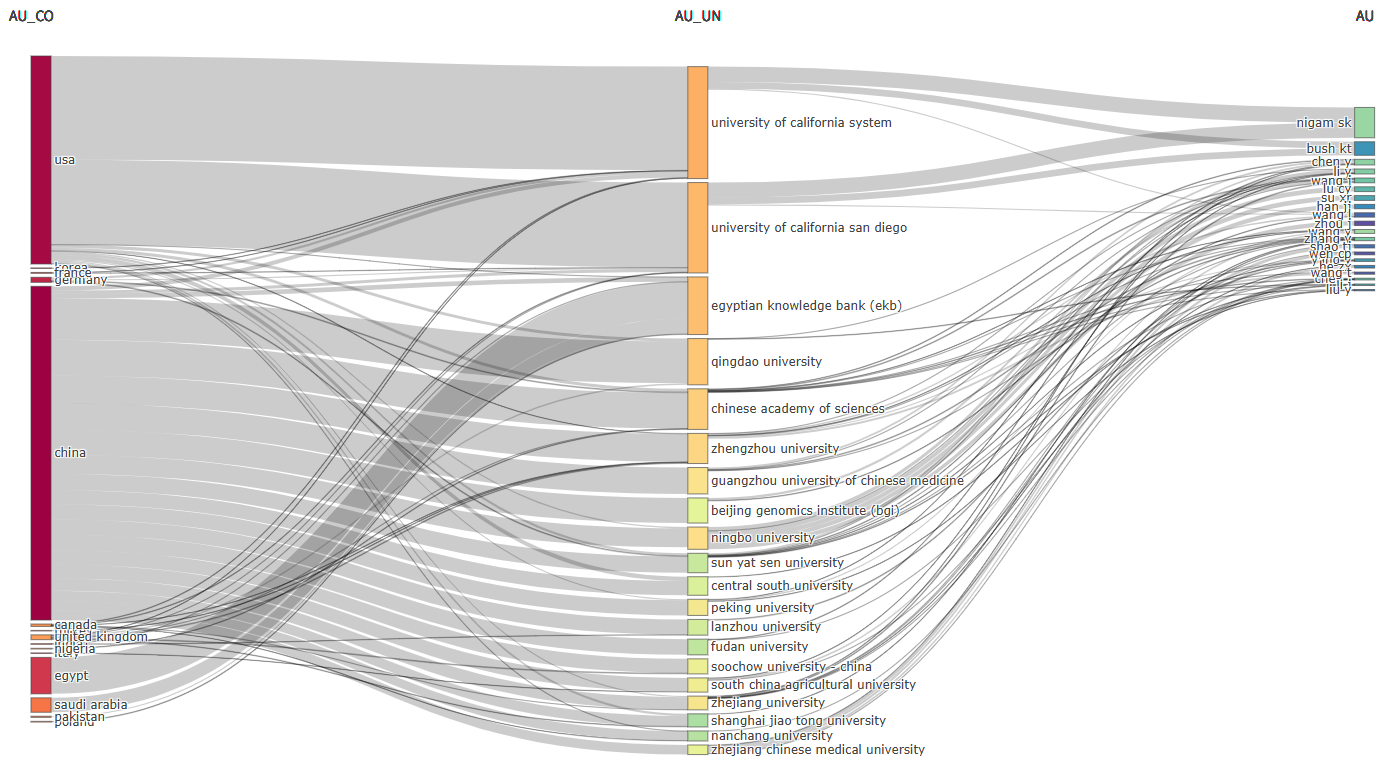
.

Supplementary Figure 1. The associations map among countries, affiliations, and authors.


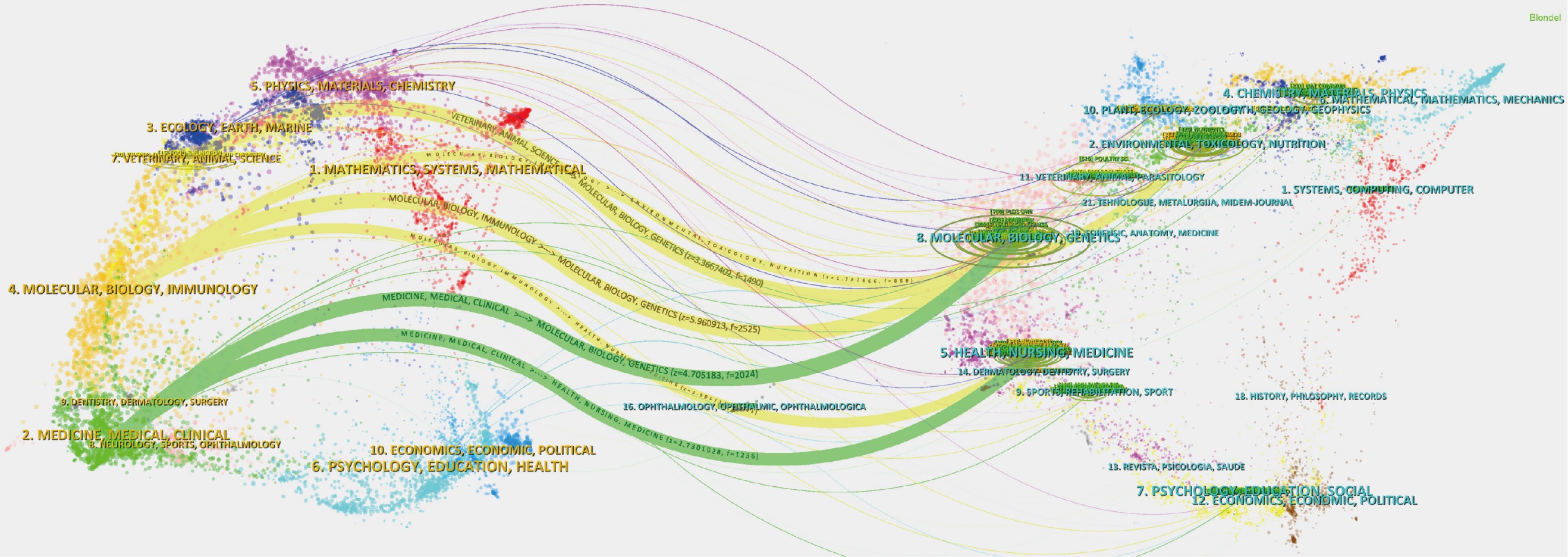


Supplementary Figure 2. Dual-map overlay of journals. The citing journals (with a minimum of 10 publications) are displayed on the left side, whereas the cited journals (with a minimum of 10 citations) are displayed on the right side of the dual-map journal overlay. The colored lines represent the connections between these journals' citations.

Supplementary Table 1. Gut microbiota composition in patients with HUA and gout

| **Object of study** | **Increased flora or metabolites compared to healthy** | **Decreased flora or metabolites compared to healthy** | **References** |
| --- | --- | --- | --- |
| 90 gout patients and 94 healthy individuals | - | *Clostridium* | 25344643 |
| 35 patients with gout and 33 healthy individuals | *Bacteroides xylanisolvens* and *Bacteroides caccae*; xanthine dehydrogenase | *Bifidobacterium pseudocatenulatum* and *Faecalibacterium prausnitzii*; allantoinase | 26852926 |
| 26 patients with gout and 26 healthy individuals | *Bacteroides*, *Porphyromonadaceae Rhodococcus*, *Erysipelatoclostridium* and *Anaerolineaceae*; alanine, glycine, taurine, succinate, α-xylose, acetate, α-glucose, and β-glucose | valine, asparagine, aspartate, citrulline, phenylalanine, and α-ketoisocaproate | 28270806 |
| 33 gouty stones patients, 25 nongouty stones, and 53 healthy individuals | *Akkermansia,Bacteroides, Ruminococcus_gnavus_group genera, Phascolarctobacterium, the Escherichia-Shigella genus* and *Proteobacteria phylum* | *Bifidobacterium, Butyricicoccus, Oscillobacter, Ruminococcaceae_UCG_010, Ruminococcaceae_UGC_013, Ruminococcus_1, Haemophilus, Clostridium_sensu_stricto_1,* and *Lachnospiraceae_ND2007_group* | 34030623 |
| A total of 102 patients with gout and 86 healthy control subjects | *Bacteroidetes,* *Fusobacteria, Prevotella* | *Proteobacteria, Escherichia spp., Klebsiella spp., Enterobacter spp. and Citrobacter spp.,* and *butyrate-producing species* | 34373464 |
| 38 treated gout patients, 38 untreated gout patients and 26 healthy individuals | *Faecalibacterium, Lachnospiraceae Clostridium, Roseburia, Cytophaga, Clostridiaceae Clostridium, Alistipes, Butyricicoccus, Pseudomonas, Ruminococcaceae,* and *Clostridium*; thymine | *Bifidobacterium, Paracoccus, Aeromonas,* and *Enterococcus*; carbohydrates | 34509383 |
| 45 asymptomatic HUA patients and 45 healthy individuals | *Alistipes, Dialister, Roseburia, Gemmiger,* and *Faecalibacterium* | *Bifidobacterium, Klebsiella,* and *Clostridium* | 34590550 |
| 204 HUA patients and 204 healthy individuals | - | *genus Coprococcus* | 34725964 |
| 20 acute gout patients | *Enterobacteriaceae* and *genus Shigella* increased compared to the recovery state | *Prevotellaceae, genus Prevotella*; acetate, propionate, and butyrate reduced compared to recovery | 35183234 |
| Forty children aged 5-15 with HUA and 45 healthy children in the same age group | *Actinomyces, Morganella,* and *Streptococcus* | the producers of SCFAs, such as *Alistipes, Faecalibacterium, and the sulfidogenic bacteria Bilophila,* and *Oscillospira* | 35574004 |
| 117 UA kidney stones patients, 123 gout patients, and 135 healthy individuals | *Bacteroides* and *Fusobacterium* | - | 35662733 |
| 36 asymptomatic HUA patients, 67 gout patients and 59 healthy individuals | Bacteria of the *Prevotella genus* compared to the control group; Individuals with AH had a higher abundance of bacteria that produce butyrate and propionate compared to those with gout, offering anti-inflammatory substances that may help prevent gout flare-ups | - | 36201123 |
| 28 gout patients with low UA levels, 20 with high levels and 17 healthy individuals | *genus Prevotella* | *genus Bifidobacterium* | 36422845 |

Supplementary Table 2. Mechanisms of Probiotic Therapy for HUA.

| **Probiotics** | **Facilitating role** | **Inhibition** | **General Mechanism** | **References** |
| --- | --- | --- | --- | --- |
| *Lacticaseibacillus rhamnosus Fmb14* and *Lacticaseibacillus rhamnosus 1155* | 1. Flora: *Prevotellaceae_UCG_001* and *Bifidobacterium*;  2. Protein expression: ABCG2 | 1. Flora: *Ruminococcaceae_UCG_014*;  2. Protein and factor expression: XOD in the liver and serum, IL-1β, and LPS | Anti-inflammatory; inhibits XOD activity; regulates UA transport protein; regulates intestinal flora | 36245501 |
| *Lacticaseibacillus rhamnosus Fmb14* | 1. Flora: diversity of intestinal flora and *Prevotella*;  2. Metabolite: SCFAs;  3. Protein and factor expression: ABCG2, ZO-1, and Occludin expression in the colon | 1. Flora: *Ruminococcus* and *Suterella*;  2. Protein and factor expression: XOD in the liver and URAT1 in the kidney, IL-1β, IL-18, LPS and TNF-α | Anti-inflammatory; inhibits XOD activity; regulates UA transport protein; produces SCFAs; regulates intestinal flora | 35843568 |
| *Lactobacillus plantarum LLY-606* | 1. Flora: *Firmicutes*, *Proteobacteria*, *Verrucomicrobia, Acidobacteria,* and *Deferribacteres*;  2. Metabolite: SCFAs;  3. Protein and factor expression: OAT1, OCT1, OCTN2, ABCG2, and Occludin | 1. Flora: *Bacteroidetes* and *Actinobacteria*;  2. Protein and factor expression: URAT1, GLUT9, OAT4, IL-1β, TNF-α, Caspase-1, COX-2, NLRP3, XOD, LPS, UA, CRE, BUN, ALT, and AST | Anti-inflammatory; inhibits XOD activity; regulates UA transport protein; produces SCFAs; regulates intestinal flora | 37264705 |
| *Lactobacillus plantarum Q7* | 1. Flora: diversity of intestinal flora, anti-inflammatory bacteria, and *Bacteroidetes/Firmicutes*; | 1. Protein and factor expression: UA, BUN, Cr, XOD, IL-1β, LPS | Anti-inflammatory; inhibits XOD activity; regulates intestinal flora | 35873427 |
| *Lactobacillus plantarum TCI227* | 1. Flora: *Lactobacillaceae* and *Ruminococcus*;  2. Metabolite: SCFAs;  3. Protein and factor expression: UUA | 1. Flora: *Deferribacteres* and *Prevotellaceae*;  2. Protein and factor expression: SUA, CRE | Produces SCFAs; regulates intestinal flora | 36432519 |
| *Lactiplantibacillus pentosus P2020* | 1. Flora: *Prevotella*;  2. Protein expression: ABCG2 in the kidney, Slc22a6, relative expression of TJ proteins (ZO1, occludin, claudin1) mRNA | 1. Flora: *Firmicutes/Bacteroidetes* ratio, *Clostridium XlVa*;  2. Protein and factor expression: GLUT9, URAT1, IL-6, TNF-α, MyD88, NF-kB p65, and Ikkβ | Anti-inflammatory; regulates UA transport protein; regulates intestinal flora | 36891165 |
| *Lactobacillus rhamnosus R31, L. rhamnosus R28-1,* and *L. reuteri L20M3* | 1. Flora: SCFA-producing intestinal flora | 1. Protein and factor expression: SUA, UUA, XOD activity levels in the liver and LPS | Anti-inflammatory; inhibits XOD activity; produces SCFAs; regulates intestinal flora | 34152353 |
| *Limosilactobacillus fermentum GR-3* | 1. Flora: *Prevotella, Intestinimonas,* and*Ruminococcus 2* | 1. Flora: *Escherichia/Shigella* and *Enterococcus*;  2. Protein and factor expression: UA, Cr, BUN, DAO, TNF-α, IL-6, and D-Lac | Anti-inflammatory; regulates intestinal flora | 36248735 |
| *Lactobacillus brevis DM9218* | - | 1. Protein and factor expression: SUA, XOD activity, inflammatory factors, and LPS | Anti-inflammatory; inhibits XOD activity; regulates intestinal flora | 30852460 |
| *Lactobacillus gasseri LG08* and *Leuconostoc mesenteroides LM58* | 1. Flora: *Firmicutes, Alistipes,* and *Bifidobacterium*;  2. Protein and factor expression: glutathione peroxidase（GSH-PX）, malonaldehyde（MDA） | 1. Flora: *Bacteroidetes, Firmicutes/Bacteroidetes* ratio;  2. Protein and factor expression: BUN, CRE, UA, UA, and IL-17 | Anti-inflammatory; regulates intestinal flora; antioxidant | 37378287 |
| *Limosilactobacillus fermentum JL-3* | 1. Flora: *Firmicutes, Alloprevotella, Oscillibacter,*and*Acidobacteria* | 1. Flora: *Bacteroidetes, Erysipelatoclostridium,* and *Mucispirillum*;  2. Protein and factor expression: IL-1β, MDA, CRE, and BUN | Anti-inflammatory; regulates intestinal flora; antioxidant | 33764849 |

**Supplementary Table 3.** Active ingredients of TCM targeted gut microbiota Therapy for HUA.

| Active ingredient category | Active ingredients of TCM | Representative herbs | Subjects | Mechanisms | Target microbiota | PMID |
| --- | --- | --- | --- | --- | --- | --- |
| Polyphenols | Resveratrol | Polygonum cuspidatum Sieb. et Zucc. | HUA mice (in vivo) | ↓SUA, CRE, BUN; ↓hepatic XOD activity; ↓intestinal UA, fecal UA; ↑intestinal UA degradation; ↓fecal LPS, ↓serum LBP, ↓renal pro-inflammatory factors (IL-6, IL-1β, TNF-α) | ↑*Lactobacillaceae*, *Lactobacillales*, *Lactobacillus_sp*; ↑SCFA-producing bacteria (such as *Clostridium*, *Bifidobacterium*, and *Faecalibaculum*); ↓*Bacteroides*, *Helicobacter*, *Prevotella* | 38613119 |
|  | Chlorogenic acid | Lonicera japonica Thunb. | HUA nephropathy rats (in vivo) | ↓SUA, BUN, and CR levels; ↓oxidative stress and inflammatory response; ↓kidney fibrosis; ↓TMAO production; ↓AKT phosphorylation, PI3K, and mTOR protein expression | ↑*Bifidobacterium*; ↓promoting TMAO synthesis bacteria (*Faecalibaculum, Blautia*, *Enterococcus*, and *Holdemania*) | 35950815 |
|  | Ferulic acid | Ligusticum chuanxiong Hort | HUA rats (in vivo) | ↓Urate absorption transporters; ↓TLR4/NF-κB pathway; ↓serum UA, BUN, creatinine; ↑secretion transporters; ↓renal oxidative stress and inflammation; remodeling gut microbiota | ↑Beneficial bacteria (*Lactobacillus*, *Ruminococcus*); ↓pathogenic bacteria (*Bacteroides*) | 36722874 |
|  | Curcumin | Curcuma Longa L. | HUA nephropathy rats (in vivo) | ↓SUA, Cr, BUN; ↓renal pathological lesions; ↓metabolic endotoxemia; ↑tightly linked proteins expression; ↓TLR4/NF-κB signaling pathway; ↓inflammation | ↑SCFA-producing bacteria (*Lactobacillus*, *Ruminococcaceae*); ↓opportunistic pathogens (*Escherichia-Shigella*, *Bacteroides*) | 34187292 |
|  | Punicalagin | Punica granatum L. | HUA mice (in vivo) | ↓SUA levels; ↑UA excretion genes (ABCG2, OAT1); ↓UA resorption gene (URAT1); ↓inflammatory cytokines (IL-1β, IL-6, TNF-α); ↓MAPK/NF-κB pathway activation; ↓renal glycometabolism disorder; ↑gut microbiota diversity | ↑SCFA-producing bacteria (*Prevotellaceae_UCG-001*, *Muribaculaceae*); ↓pro-inflammatory bacteria (*Parabacteroides*, *Oscillibacter*, *Desulfovibrio*, *Tuzzerella*) | 38609050 |
| polysaccharides | Inulin | Cichorium intybus L. | KO (Uox-knockout) and WT (wild-type) mice | ↓SUA; ↑intestinal tight junction proteins (ZO-1, occludin); ↑ABCG2 expression in intestine; ↓hepatic XOD activity; ↓serum levels of uremic toxins (IS, PCS); ↑microbial diversity; ↑production of SCFAs (acetate, propionate, butyrate) | ↑SCFA-producing bacteria (*Akkermansia*, *Ruminococcus*) | 33104864 |
|  | Enteromorpha prolifera polysaccharide | Chlorophyta | HUA mice (in vivo) | ↓SUA, BUN, serum XOD, hepatic XOD; ↑UA excretion genes (ABCG2, OAT1, NPT1); ↓UA resorption gene (URAT1); improved renal histopathology; maintained intestinal flora stability | ↑*Parasutterella* | 34687833 |
| Flavonoids | Quercetin | Taxillus chinensis（DC.）Danser | HUA mice (in vivo) | ↓SUA, CRE, BUN; ↓liver XOD activity; ↓IL-1β; ↑degradation of purine nucleosides; ↓expression of UA production enzymes (NT5E, ADA, PNP); ↑renal UA excretion genes (OAT3) | ↑*Lactobacillus aviarius CML180* | 37717681 |
|  | Myricetin-Nobiletin Hybrid (MNH) | Waxberry, Citrus reticulata Blanco | HUA mice (in vivo) | ↓SUA, CRE, BUN; repairs renal damage; regulates metabolic pathways (glycerophospholipid, arachidonic acid, alanine metabolism) | ↑*Norank_f_Muribaculaceae*; ↓*Lactobacillus*, *Limosilactobacillus* | 37465570 |
|  | Fisetin | Rhus succedanea L | HUA nephropathy mice (in vivo) | ↓SUA, CRE, BUN; ↓ renal fibrosis; ↓AHR activation modulates gut microbiota-mediated tryptophan metabolism | ↑*Epsilonbacteraeota***; ↓***Firmicutes* | 34505780 |
|  | Flavonoid extract of saffron | Crocus sativus L. | HUA rats (in vivo) | ↓SUA, CRE, BUN; ↓hepatic XOD protein & XDH mRNA; ↓renal IL-1β; ↑degradation of purine nucleosides; ↑lipid metabolites (LysoPCs, SMs, Cer); ↑aspartic acid, ↓betaine; ↓MDA levels,↑GSH-PX/SOD activities | ↑Beneficial (*Roseburia*, *Clostridium_sp.*, *Gastranaerophilales*); ↓Pathogenic (*Alloprevotella*, *Parabacteroides_goldsteinii*, *Clostridioides*, *Holdemania*, *Erysipelatoclostridiaceae*, *Erysipelatoclostridium*) | 36482025 |
| Alkaloids | Berberine | Coptis chinensis Franch. | HUA rats (in vivo) | ↓XOD; ↑colon urate transport ABCG2; ↓colon urate transport Galectin-9 | ↑*Lactobacillus*; ↓*Bacteroidetes* | 36424788 |
|  | Nuciferine | Nelumbo nucifera Gaertn. | HUA rats (in vivo) | ↓XOD; ↑UA excretion genes (OCT1, OAT1); ↓UA resorption gene (GLUT9, URAT1); ↓inflammatory cytokines (IL-1β); restores TCA cycle (Pyruvate ↓, Succinate ↑); modulates glycerophospholipids (LysoPC(18:2) ↑, PC(18:0/20:4) ↓) | ↑Beneficial (*Firmicutes*); ↓ *Escherichia-Shigella*, Conditionally pathogenic genera | 33146009 |

Table Notes: ↑: Upregulation or increase in the specified factor or component. ↓: Downregulation or decrease in the specified factor or component.
